# Cytotoxic granule secretion by lymphocytes and its link to immune homeostasis

**DOI:** 10.12688/f1000research.6754.1

**Published:** 2015-09-30

**Authors:** Geneviève de Saint Basile, Fernando E. Sepulveda, Sophia Maschalidi, Alain Fischer

**Affiliations:** 1 INSERM UMR1163, Laboratory of Normal and Pathological Homeostasis of the Immune System, Paris, F-75015, France; 2 Paris Descartes University-Sorbonne Paris Cité, Imagine Institute, Paris, F-75015, France; 3 Centre d’Etudes des Déficits Immunitaires, Assistance Publique-Hôpitaux de Paris, Assistance Publique-Hôpitaux de Paris, Paris, France; 4 Immunology and Pediatric Hematology Department, Necker Children’s Hospital, AP-HP, Paris, France; 5 Collège de France, Paris, F-75005, France

**Keywords:** haemophagocytic lymphohistiocytosis, cytotoxic, natural killer lymphocytes

## Abstract

The granule-dependent cytotoxic activity of T and natural killer lymphocytes has progressively emerged as an important effector pathway not only for host defence but also for immune regulation. The analysis of an early-onset, severe, primary immune dysregulatory syndrome known as hemophagocytic lymphohistiocytosis (HLH) has been decisive in highlighting this latter role and identifying key effectors on the basis of gene mutation analyses and mediators in the maturation and secretion of cytotoxic granules. Studies of cytotoxicity-deficient murine counterparts have helped to define primary HLH as a syndrome in which uncontrolled T-cell activation in response to lymphocytic choriomeningitis virus infection results in excessive macrophage activation and inflammation-associated cytopenia. Recent recognition of late-onset HLH, which occurs in a variety of settings, in association with hypomorphic, monoallelic mutations in genes encoding components of the granule-dependent cytotoxic pathway or even in the absence of such mutations has broadened our view about the mechanisms that underlie the perturbation of immune homeostasis. These findings have led to the development of a model in which disease occurs when a threshold is reached through the accumulation of genetic and environmental risk factors. Nevertheless, validation of this model will require further investigations.

## Granule-dependent cytotoxic activity is a key regulator of immune homeostasis

The role of cytotoxic lymphocytes in defending the organism against virally infected cells and tumor cells has long been recognized. Through the polarized secretion of granules containing cytotoxic proteins, cytotoxic lymphocytes can rapidly kill their cognate target cells ^1^. However, only recently have studies of inherited deficiencies of lymphocyte cytotoxic activity in humans highlighted the importance of lymphocyte cytotoxicity in the resolution of inflammation ^2^.

Hemophagocytic lymphohistiocytosis (HLH) syndrome is a life-threatening immune dysregulation condition characterized by an excessive inflammatory response and hypercytokinemia. It is generally triggered by an infective agent, such as the members of the human herpes virus family. HLH manifests as the massive expansion and activation of polyclonal CD8 ^+^ T cells; this probably results from the failure of cytotoxic T lymphocyte (CTL) and natural killer (NK) cells to clear antigen-presenting cells (APCs) and therefore terminate an immune response ^3, 4^. Uncontrolled T-cell activation leads to macrophage activation, a pro-inflammatory cytokine storm, cytopenia, coagulopathy, multi-organ cellular infiltration, and organ dysfunction ^5– 7^. The link between cytotoxicity and lymphocyte homeostasis was first demonstrated 15 years ago, following the identification of perforin deficiency in a subgroup of patients with an inherited form of HLH (familial HLH, or FHL) ^2^. Undoubtedly, this step was decisive in the characterization of the other causes of inherited HLH and in the identification of key effectors that mediate the exocytic machinery in cytotoxic lymphocytes ^8, 9^. Naturally occurring or engineered mice with a similar cytotoxicity defect have proven to be very useful tools for further understanding the underlying pathophysiological mechanism ^4, 10, 11^. The observation that a relatively mild cytotoxic defect can be associated with defective immune surveillance or atypical HLH onset or both has now raised the question of the underlying individual’s risk factors that are associated with a subtle cytotoxic defect to drive disease onset.

## Studies of inherited defects of cytotoxicity have revealed critical effectors of cytotoxic granule exocytosis

The sequence of events by which T/NK cytotoxic lymphocytes kill targets is now fairly well characterized. When cytotoxic lymphocytes recognize their cognate target cells, they form a transient cellular conjugate and an immunological synapse (IS) at the area of cell-cell contact ^1, 12^. Within the very first minutes of target cell interaction, the actin network, which was previously positioned across the entire contact area, is progressively depleted from the center of the synapse, as recently highlighted by the use of rapid, super-resolution imaging methods ^13– 15^. The microtubule-organizing centre (MTOC) rapidly moves toward the target cell, while the cytotoxic granules (containing the cytotoxic proteins perforin and granzymes) migrate along microtubules and cluster around the MTOC ^8, 16^. At the IS, the centrosome can touch the membrane and then deliver polarized cytotoxic granules ^17^. The granules fuse with the presynaptic membrane and secrete their contents into the synaptic cleft. This accurate, polarized secretion of lytic reagents ensures that cytotoxic cells destroy only the bound target cell and not bystander cells. Within the synaptic cleft, perforin oligomerizes, creates pores in the target cell membrane, and thus enables the pro-apoptotic granzymes to access the target cytosol ^18– 20^. The mechanism of granzyme uptake has long been subject to debate. By using time-lapse microscopy techniques that pinpoint the moment at which perforin permeabilizes the target cell plasma membrane within the IS, researchers observed that the time course of target cell apoptosis after pore formation is very rapid (that is, within 10 mins) ^21, 22^. In contrast to what has been proposed in other studies ^23– 25^, this suggests that granzymes cross the plasma membrane and are not taken up in endosomes.

In addition to perforin deficiency ^2^, which accounts for about one third of the FHL cases, several inherited forms of HLH are characterized by failure to deliver cytotoxic granule contents. The identification of the underlying molecular causes has contributed to our understanding of the key steps in the secretion of cytotoxic granules at the IS ^8^. Biallelic mutations in *UNC13D* (encoding Munc13-4, accounting for about one third of FHL cases), *STX11* (encoding syntaxin 11, about 5% of FHL cases), and *STXBP2* (encoding syntaxin-binding protein 2, also known as Munc18-2, about 20% of FHL cases) led to the occurrence of HLH in FHL types 3, 4, and 5, respectively ^26– 29^. In about 10% of FHL cases, the molecular defect remains uncharacterized. Biallelic mutations in *RAB27A* (encoding the small GTPase Rab27a) and *LYST* (encoding lysosomal trafficking regulator) account for the development of HLH in Griscelli syndrome ^30^ and Chédiak-Higashi syndrome ^31^, respectively. Remarkably, each of these molecules mediates a discrete, non-redundant step in cytotoxic granule exocytosis at the IS. Rab27a and Munc13-4 are respectively required for the granule docking and priming steps at the plasma membrane, whereas syntaxin 11 interacts with Munc18-2 to enable granules to fuse with the plasma membrane ^8^. Although the role of LYST is less well understood, it may regulate a late granule maturation step ^32^. The effector molecules’ partners and interconnections have been progressively characterized to reveal the overall picture of granule exocytosis. Notably, interaction between Rab27a and Munc13-4 was shown to be mandatory for tethering the cytotoxic granules at the IS in order to complete the exocytic process ^33^. Munc13-4 interacts with several syntaxin isoforms, among them syntaxin 11 ^34^. Furthermore, Rab27a binds to three different members of the synaptotagmin-like (SLP1-3) family expressed in cytotoxic cells that have partially overlapping functions in granule transport and docking ^35– 37^.

It has been proposed that direct contact between polarized centrosomes and the plasma membrane drives cytotoxic granule delivery at the IS ^17, 38^. However, there is evidence to suggest that alternative mechanisms are involved, such as the observation that very rapid, effective cytotoxic granule secretion can precede MTOC polarization in some CTL-target cell conjugates ^39^. In the latter study, inhibition of MTOC polarization did not prevent cytotoxic granule release. Furthermore, the Slp3/Rab27a complex expressed in cytotoxic cells was shown to interact with a kinesin motor and to mediate the terminal transport of polarized cytotoxic granules toward the IS ^35^. In view of the diversity of *in vivo* settings in which cytotoxic cells are triggered, one can legitimately hypothesize that granule delivery may occur via several different routes. Indeed, recent research has shown that cytotoxic cells are heterogeneous and change their killing performance over time and as a function of antigenic stimulation ^40, 41^. Timescale studies of single NK cells or CTLs have revealed a progressive increase in the rapidity and efficiency of killing during serial killing, which also varies according to the avidity of antigen recognition ^40, 41^. It remains not well understood how what has been shown *in vitro* applies *in vivo*, such as the nature of target cells and the strength of triggering signal.

## The use of animal models to characterize the pathophysiology of hemophagocytic lymphohistiocytosis

Animal models of primary HLH in which cytotoxicity-deficient mice are challenged with a virus have proven to be invaluable for understanding the pathogenesis of HLH under defined conditions. It has been demonstrated that after lymphocytic choriomeningitis virus (LCMV) infection of perforin-deficient mice, hyperactive CTLs and high levels of interferon-gamma (IFN-γ) are the driving forces behind the development of fatal HLH ^4^. LCMV’s potent induction of HLH might be due, at least in part, to its ability to infect APCs and thus strongly stimulate a T-cell response without the need for antigen cross-presentation. Likewise, Epstein-Barr virus (EBV), a major trigger of HLH in humans, can directly infect B cells, which also have an antigen presentation function and trigger prolonged antigenic stimulation when not eliminated by cytotoxic lymphocytes. In addition to viral priming, antigen persistence and prolonged presentation were shown to be critical in the development of primary HLH in murine models ^4, 10^. This appears to contrast with the onset of primary HLH in newborn infants or even fetuses with cytotoxicity defects, since a pathogen trigger cannot be identified in many cases ^42, 43^. Although as-yet-unknown microorganisms may act as the trigger, this observation suggests that, in contrast to the situation in mice, the granule-dependent cytotoxic pathway in humans also has a role in T-cell homeostasis in the absence of an external stimulus (as is also the case for the Fas/FasL pathway). It has been shown that the elimination of a rare, antigen-presenting dendritic cell (DC) population by CD8 ^+^ T cells in a negative feedback loop is a critical determinant of the magnitude of T-cell responses ^44, 45^. Thus, the elimination of specific APC populations probably determines the activation status and survival of hyper-reactive T cells and acts as a rheostat by limiting T-cell responses. Whether the granule-dependent cytotoxic pathway is also participating to check self-reactive T/B cells is therefore a possibility that needs to be further investigated.

**Figure 1.**
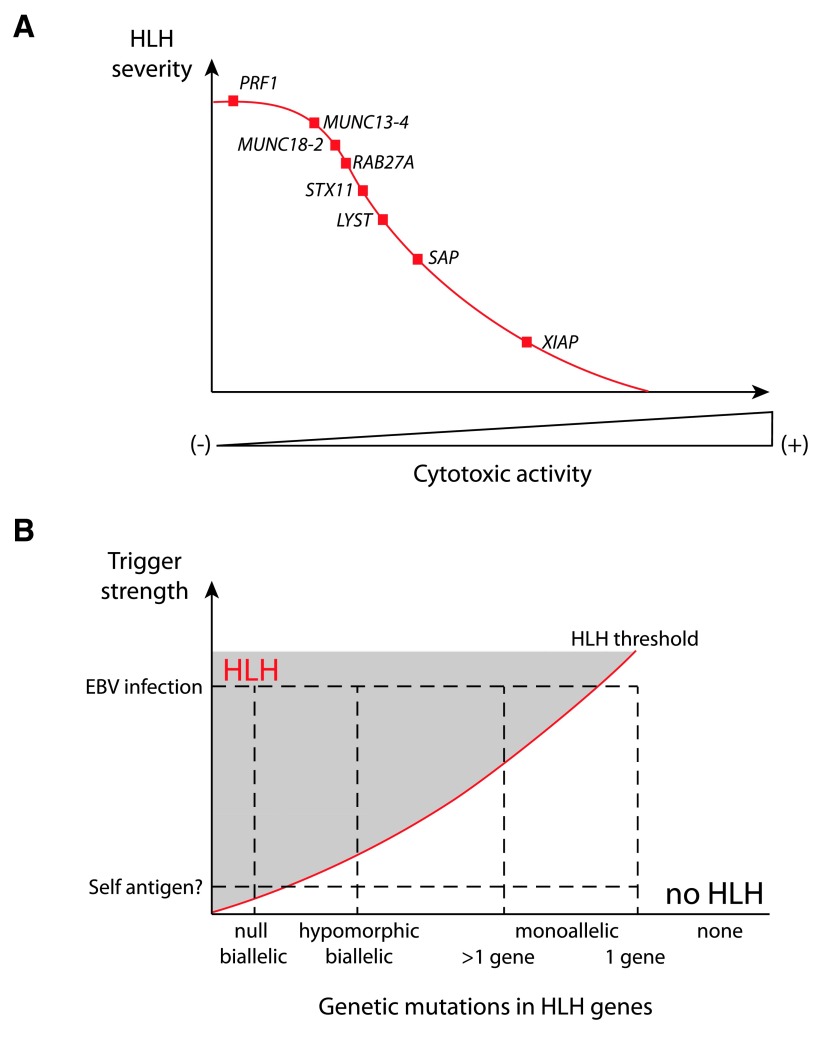
Impact of various genetic and environmental risk factors on threshold of hemophagocytic lymphohistiocytosis (HLH) development. ( **A**) A gradient of hemophagocytic lymphohistiocytosis (HLH) severity correlates with the defect in cytotoxic activity of lymphocytes that results from various genetic defects in humans and mice. Null mutations are considered in this image. ( **B**) Evolving view of the risk factors inducing HLH development. Mild to extreme immune stimuli, in combination with severe (null) mutations, hypomorphic mutations, monoallelic mutation in several or one of the genes involved in HLH, appear to determine an individual’s risk for developing HLH. HLH risk lies above the red line in the hatched area.

In mice, the degree of cytotoxicity impairment appears to be the best predictor of the development and severity of HLH, as shown by studies of the time course of HLH onset in various HLH-prone strains with defects in the granule-dependent cytotoxic pathway ^10, 46^. The same is true in humans ^10, 46^ ( Figure 1A). In genetically determined murine models of HLH, the cytotoxicity of both T cells and NK cells is impaired. However, in contrast to CD8 ^+^ T-cell depletion, NK cell depletion in perforin-deficient mice did not prevent the development of manifestations of HLH ^4^. CTLs were thus considered to be the main players in the development of HLH. However, recent work has revealed that T cells and NK cells have a non-redundant cytotoxic function in HLH: CTLs mediate LCMV viral clearance, whereas NK cells limit hyperactivation of CTLs ^11^. This finding further suggests that the perforin-dependent cytotoxic activity of NK cells has a key role in the maintenance of immune homeostasis and the prevention of immunopathology ^47, 48^. However, the underlying mechanism through either direct or indirect T/NK cell interactions remains to be characterized. Furthermore, one cannot fully exclude the participation of other potentially cytotoxic cells such as invariant NK T (iNKT) cells and CD4 ^+^ T-cell subsets, including specific regulatory T (Treg) populations, in this setting. It is also worth noting that in syntaxin 11-deficient mice, which display a milder cytotoxic defect and less severe HLH than perforin-deficient mice, blockade of inhibitory receptors of T-cell exhaustion (such as PD1/PDL1) dramatically increases the severity of HLH and results in fatal disease ^49^. This finding indicates that T-cell exhaustion is another important modulator of HLH severity.

The *in vivo* failure of cytotoxic cells to eliminate target cells leads to a fatal cytokine storm, a hallmark of HLH. Previous research has shown that the threshold of T-cell activation determines whether a lytic synapse (which is induced at low antigen concentrations and which enables cytotoxic activity) or a stimulatory synapse (induced at high antigen concentrations and which enables both cytotoxic activity and IFN-γ production) is formed ^50^. Remarkably, it was recently shown that cytotoxicity-deficient lymphocytes form longer contacts with their cognate target, thus resulting in many successive rounds of Ca ^2+^ flux into cytotoxic cells and triggering of pro-inflammatory cytokine secretions ^51^. Thus, the cytokine storm as observed in HLH likely depends on both quantity and quality of contacts formed between cytotoxic cells and APCs.

Phagocytosis of blood cells by macrophages (known as hemophagocytosis) is another hallmark of primary HLH, although it can be observed in a variety of infectious or inflammatory disorders ^52^. A study of perforin-deficient mice has revealed that IFN-γ specifically triggers this process, which can be reproduced in wild-type mice by inducing the sustained elevation of IFN-γ. Direct, IFN-γ-dependent activation of macrophages prompts the development of severe, consumptive anemia and other types of cytopenia, probably through direct changes in the macrophages’ endocytic uptake ^53^. These results indicate that hemophagocytosis is actually an adapted response to sustained or severe inflammation. Further details on the role of macrophages and other inflammatory cells in the pathophysiology of HLH have been provided in recent reviews ^5, 6, 54^.

Although cytotoxic lymphocytes exert a key role in the development of primary HLH, other immune cells and signaling pathways may also contribute. It has been shown that MyD88, which mediates Toll-like receptor (TLR) and interleukin-1 (IL-1) signaling, is required for HLH development in Unc13d-deficient mice, suggesting that innate immune cells contribute to the development of HLH ^55^. Moreover, high levels of IL-4 or repeated TLR9 stimulation in wild-type mice can induce the development of an HLH-like syndrome ^56, 57^. Hence, proteins from the cytotoxic exocytic pathway may have additional functions in other immune cell types (such as inflammatory cells), the absence of which modulates the pathogenesis of HLH. More generally, any regulatory molecule involved in an inflammatory pathway might contribute to the development of the manifestations of HLH.

## What is the minimum level of cytotoxic activity required to preserve immune homeostasis?

Genetically determined forms of HLH can occur even when a cytotoxicity defect is only partial or apparently absent. This is the case in X-linked lymphoproliferative syndrome (XLP). Patients with XLP are extremely vulnerable to EBV infection and most go on to develop HLH ^58^. There are two genetic forms: XLP-1 and XLP-2. Firstly, XLP-1 results from a deficiency in the signaling lymphocyte activation molecule (SLAM)-associated protein (SAP) ^59– 61^. SAP-deficient CTLs and NK cells are selectively impaired in their cytotoxic response to infected B cells; the response requires interaction between SLAM family receptors and subsequent SAP-dependent signaling in T lymphocytes but not in other cell types ^62, 63^. Secondly, XLP-2 results from a deficiency in the X-linked inhibitor of apoptosis protein (XIAP) (also known as BIRC4) ^64^. However, XIAP-deficient CTLs and NK cells exhibit apparently normal *in vitro* cytotoxic responses (regardless of the SLAM-receptor dependency). The cytotoxic activity of iNKT cells is known to be activated by EBV-infected B cells ^65^. Indeed, the exacerbated apoptosis of XIAP-deficient iNKT cells, induced by EBV infection, might be involved in the development of HLH in XLP-2. Alternatively, the mechanisms underlying EBV-driven HLH in XLP-2 may differ completely from those observed in XLP-1 and other inherited forms of HLH. In a setting of XIAP deficiency, the accumulation of apoptotic cells and the persistence of EBV-infected cells might trigger abnormal inflammation and contribute to the development of HLH. This hypothesis is supported by the observation that XIAP deficiency in mice results in excessive DC death and inflammasome activation ^66^.

It is difficult to assess the minimal level of cytotoxic activity required for the maintenance of immune homeostasis. Hypomorphic mutations in HLH genes that preserve residual cytotoxicity significantly delay the onset of HLH but predispose patients to hematological cancers ^67– 69^. Adult patients with HLH have been found to carry a monoallelic mutation in one or more FHL genes ^70– 73^. These findings suggest that the accumulation of heterozygous mutations that partially impair the granule-dependent cytotoxic pathway may have an additional functional impact. This hypothesis could be tested by studying inter-crossed animal models of HLH with monoallelic mutations. The concept whereby a monoallelic mutation in cytotoxicity-related genes can lead to immune disturbance also requires far more investigation, particularly in much larger cohorts of patients with induced HLH versus healthy controls. When the cytotoxicity defect is mild, the relative weight of additional genetic and environmental factors in HLH triggering is probably greater. It is tempting to speculate that (i) “extreme” stimuli may be sufficient to induce sporadic HLH development in any individual and (ii) the overall risk is augmented by the accumulation of genetic variants promoting excessive or poorly regulated immune response ( Figure 1B). Along these lines, mutations in genes controlling inflammatory processes may also contribute to HLH. Recently, *de novo* activating mutations in the nucleotide-binding domain of inflammasome component *NLRC4*, associated with high levels of inflammatory cytokines in general and of IL-18 in particular, were found to be linked to recurrent HLH ^74– 76^. Since impaired cytotoxicity was not detected in that setting, this finding highlights the role of additional molecules in the pathophysiological process leading to HLH. By coupling next-generation sequencing to animal model studies, it should now be possible to determine whether HLH can be a polygenic condition in adults.

## Concluding remarks

Over the last few decades, characterization of the molecular bases of primary HLH has highlighted the critical role of CTL activity in the control of immune homeostasis and has identified key effectors of cytotoxic granule exocytosis and their specific functions along the cytotoxic pathway. Broader knowledge of the scope of HLH occurrence has prompted the hypothesis whereby HLH is a “threshold” disease. A combination of both genetic factors and environmental factors (infections, self-antigens, and so on) is needed for the development of HLH in a context of residual cytotoxicity. Some cases of HLH do not appear to be directly related to a cytotoxicity defect, indicating that other genes, notably involved in macrophage-related inflammation, regulating the same disease pathway also have a role. Characterizing the synergistic connections between the various risk factors for HLH will be a key challenge in the coming years.

## Abbreviations

APC, antigen-presenting cell; CTL, cytotoxic T lymphocyte; DC, dendritic cell; EBV, Epstein-Barr virus; FHL, familial hemophagocytic lymphohistiocytosis; HLH, hemophagocytic lymphohistiocytosis; IFN-γ, interferon-gamma; IL, interleukin; iNKT, invariant natural killer T; IS, immunological synapse; LCMV, lymphocytic choriomeningitis virus; MTOC, microtubule-organizing centre; NK, natural killer; SAP, signaling lymphocyte activation molecule-associated protein; SLAM, signaling lymphocyte activation molecule; TLR, Toll-like receptor; XIAP, X-linked inhibitor of apoptosis protein; XLP, X-linked lymphoproliferative syndrome.
