# Supplementary material for: Cytotoxic granule secretion by lymphocytes and its link to immune homeostasis
Source: F1000Res. 2015 Sep 30;4(F1000 Faculty Rev):930. [Version 1] doi: 10.12688/f1000research.6754.1 (PMC4648190; doi:10.12688/f1000research.6754.1)
Supplement: Peer review history

# Peer review: Referee response for version 1

**DOI:** 10.5256/f1000research.7256.r10629

**Published:** 2015-09-30

**Authors:** Ilia Voskoboinik

**Affiliations:** 1 Cancer immunology Program, Peter MacCallum Cancer Centre, East Melbourne, Australia

I have read this submission. I believe that I have an appropriate level of expertise to confirm that it is of an acceptable scientific standard.

# Peer review: Referee response for version 1

**DOI:** 10.5256/f1000research.7256.r10627

**Published:** 2015-09-30

**Authors:** Loïc Dupré

**Affiliations:** 1 Centre de Physiopathologie de Toulouse Purpan, INSERM, UMR1043, Toulouse, France

I have read this submission. I believe that I have an appropriate level of expertise to confirm that it is of an acceptable scientific standard.
